# Nerve injury augments *Cacna2d1* transcription *via* CK2-mediated phosphorylation of the histone deacetylase HDAC2 in dorsal root ganglia

**DOI:** 10.1016/j.jbc.2024.107848

**Published:** 2024-09-30

**Authors:** Krishna Ghosh, Yuying Huang, Shao-Rui Chen, Hui-Lin Pan

**Affiliations:** Center for Neuroscience and Pain Research, Department of Anesthesiology and Perioperative Medicine, The University of Texas MD Anderson Cancer Center, Houston, Texas, USA

**Keywords:** casein kinase II, chromatin plasticity, epigenetic, histone modification, primary sensory neuron, neuropathic pain

## Abstract

The development of chronic neuropathic pain involves complex synaptic and epigenetic mechanisms. Nerve injury causes sustained upregulation of α2δ-1 (encoded by the *Cacna2d1* gene) in the dorsal root ganglion (DRG), contributing to pain hypersensitivity by directly interacting with and augmenting presynaptic NMDA receptor activity in the spinal dorsal horn. Under normal conditions, histone deacetylase 2 (HDAC2) is highly enriched at the *Cacna2d1* gene promoter in the DRG, which constitutively suppresses *Cacna2d1* transcription. However, nerve injury leads to HDAC2 dissociation from the *Cacna2d1* promoter, promoting the enrichment of active histone marks and *Cacna2d1* transcription in primary sensory neurons. In this study, we determined the mechanism by which nerve injury diminishes HDAC2 occupancy at the *Cacna2d1* promoter in the DRG. Spinal nerve injury in rats increased serine-394 phosphorylation of HDAC2 in the DRG. Coimmunoprecipitation showed that nerve injury enhanced the physical interaction between HDAC2 and casein kinase II (CK2) in the DRG. Furthermore, repeated intrathecal treatment with CX-4945, a potent and specific CK2 inhibitor, markedly reversed nerve injury-induced pain hypersensitivity, HDAC2 phosphorylation, and α2δ-1 expression levels in the DRG. In addition, treatment with CX-4945 largely restored HDAC2 enrichment at the *Cacna2d1* promoter and reduced the elevated levels of acetylated H3 and H4 histones, particularly H3K9ac and H4K5ac, at the *Cacna2d1* promoter in the injured DRG. These findings suggest that nerve injury increases CK2 activity and CK2-HDAC2 interactions, which enhance HDAC2 phosphorylation in the DRG. This, in turn, diminishes HDAC2 enrichment at the *Cacna2d1* promoter, thereby promoting *Cacna2d1* transcription.

Chronic neuropathic pain, resulting from injury or disease of the somatosensory nervous system, remains challenging to treat. Our current knowledge of the molecular mechanisms that initiate and sustain neuropathic pain is still limited. Increased synaptic N-methyl-D-aspartate receptors (NMDARs) activity plays a predominant role in the amplification of nociceptive signals from primary sensory neurons in neuropathic pain caused by traumatic nerve injury and chemotherapy (1, 2, 3, 4, 5). Nerve injury causes early and sustained high expression of α2δ-1 (encoded by the *Cacna2d1* gene) in the dorsal root ganglion (DRG) (6, 7). Increased α2δ-1 proteins directly interact with NMDARs to promote their synaptic expression at central terminals of DRG neurons, which underlies the therapeutic effect of gabapentinoids commonly used for treating neuropathic pain (3, 8, 9, 10). Histone deacetylase 2 (HDAC2) is highly enriched at the *Cacna2d1* promoter in DRG neurons (11, 12). Although nerve injury increases the total HDAC2 protein level in the DRG (12), it paradoxically induces HDAC2 dissociation from the *Cacna2d1* promoter region, resulting in increased histone acetylation and ensuing *Cacna2d1* transcription in the DRG (11). However, little is known about how nerve injury diminishes HDAC2 enrichment at the *Cacna2d1* promoter in the DRG.

Phosphorylation of HDAC2 critically influences its activity and its ability to form DNA-protein and protein-protein complexes at the chromatin level (13, 14, 15, 16). Interestingly, phosphorylation of serine 394 (Ser^394^), Ser^422^, and Ser^424^ of HDAC2 not only affects its enzymatic activity but also impacts its interactions with other protein partners of its repressor complexes, including mammalian Sin3 (mSin3), RB binding protein 4, chromatin remodeling factor (RbAp48), CoREST, and Mi-2 (also known as chromodomain-helicase-DNA-binding protein 3, CHD3) (15, 16, 17, 18). Thus, phosphorylation of HDAC2 may act as a switch in directing the HDAC2-complex organization and dynamic acetylation-deacetylation at the bound chromatin. At present, it is unclear whether nerve injury affects HDAC2 phosphorylation in the DRG.

Casein kinase II (CK2) is a highly conserved serine/threonine protein kinase and plays a pivotal role in regulating cellular signaling processes (19). CK2 is constitutively active and composed of two catalytic (α or α′) subunits and two regulatory β subunits (19, 20), which are highly dynamic and undergoes nucleocytoplasmic trafficking (21). The α and β subunits of CK2 are abundantly expressed in the DRG and spinal dorsal horn, and CK2 inhibition reduces synaptic NMDAR activity and pain hypersensitivity induced by nerve injury (22, 23). Interestingly, the C-terminal serine residues of HDAC2 (Ser^394/422/424^) are uniquely phosphorylated by CK2 *in vitro* (16, 17). In vascular smooth muscle cells, knockdown of CK2α or expression of a dominant-negative CK2α prevents HDAC2 Ser^394^ phosphorylation and its interaction with a Krüppel-like transcription factor (24). Furthermore, CK2α overexpression elevates HDAC2 Ser^394^ phosphorylation and augments cardiac hypertrophy in mice (18). However, the potential role of CK2 in regulating HDAC2 phosphorylation and α2δ-1 expression in neuropathic pain remains elusive.

In this study, we tested the hypothesis that nerve injury-induced CK2 hyperactivity enhances HDAC2 phosphorylation and diminishes HDAC2 enrichment at the *Cacna2d1* promoter, thereby facilitating *Cacna2d1* transcription in the DRG. Our findings highlight the pivotal role of CK2 in regulating HDAC2 abundance at the *Cacna2d1* promoter and α2δ-1 expression by directly interacting with HDAC2 in injured DRGs. This new information advances our mechanistic understanding of the epigenetic processes contributing to the development of chronic neuropathic pain.

## Results

### Nerve injury increases α2δ-1 expression and HDAC2 phosphorylation in the DRG

**Figure 1:**
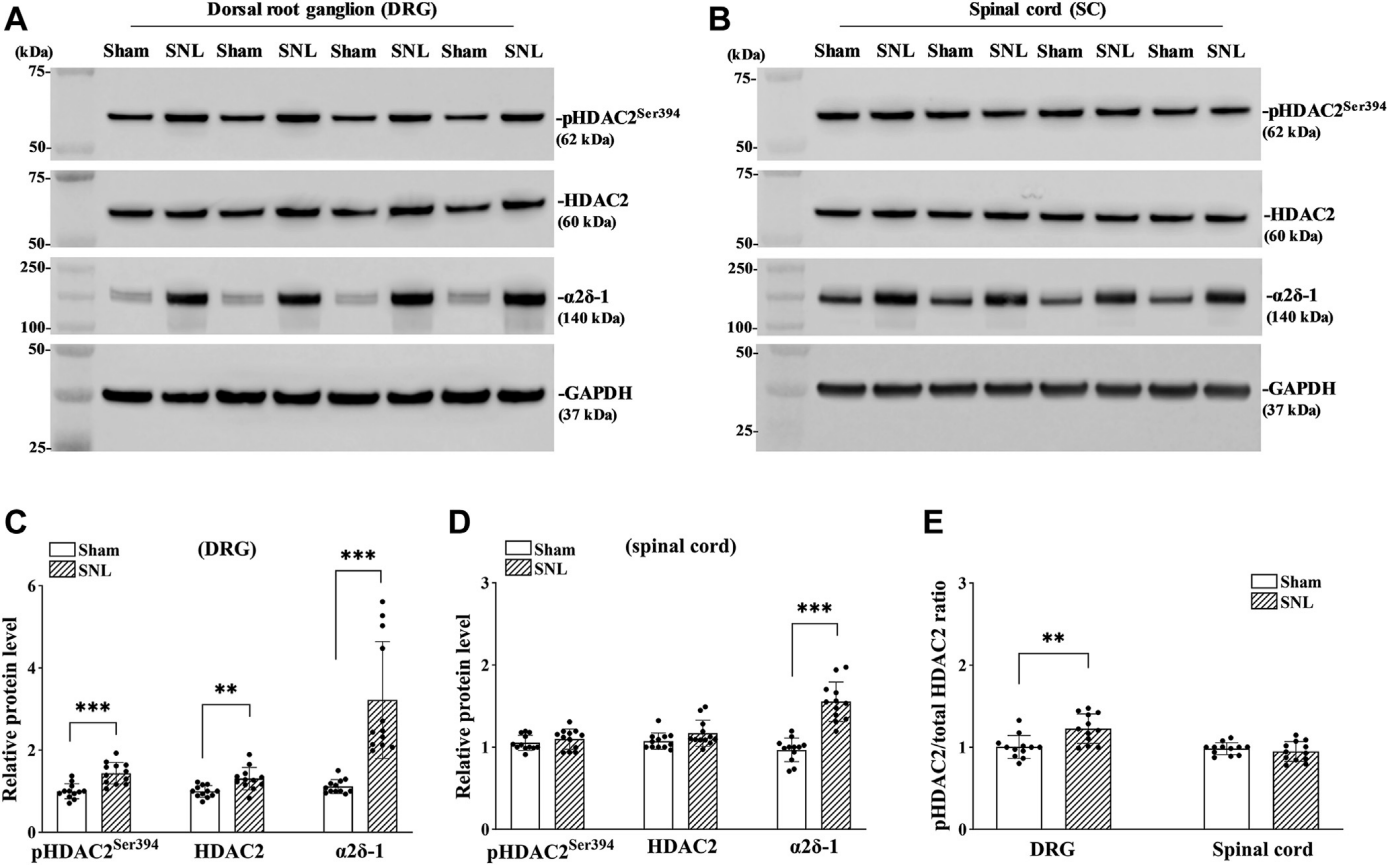
**Nerve injury increases HDAC2 phosphorylation and α2δ-1 expression in the DRG.***A–D*, Representative immunoblotting images and quantification show the protein level of phospho-serine^394^ of HDAC2 (pHDAC2^Ser394^), HDAC2, and α2δ-1 in the DRG (*A* and *C*) and dorsal spinal cord (*B* and *D*) of rats subjected to spinal nerve ligation (SNL) or sham surgery (n = 12 rats per group). Total proteins were extracted from the tissue 3 weeks after surgery. *E*, changes in the pHDAC2^Ser394^/HDAC2 ratio in the DRG and spinal cord from SNL and sham rats. ∗∗*p* < 0.01, ∗∗∗*p* < 0.001 (two-tailed Student *t* test). Data are expressed as means ± SD.

Reduced HDAC2 occupancy at the *Cacna2d1* promoter is involved in peripheral nerve injury-induced transcriptional activation of *Cacna2d1* in the DRG (11). HDAC2 is uniquely phosphorylated by CK2 (16, 17). The Ser^394^ phosphorylation of HDAC2 (pHDAC2^Ser394^) is critical in regulating its activity (16, 17). Hence, we determined whether nerve injury augments HDAC2 phosphorylation in the DRG. Rats subjected to L5 and L6 spinal nerve ligation (SNL) or sham surgery, and DRG and dorsal spinal cord tissues were obtained 3 weeks after surgery. Immunoblotting with an anti-pHDAC2^Ser394^ antibody showed that SNL significantly increased the level of phosphorylated HDAC2 at serine 394 in the DRG (n = 12 rats per group, t_(22)_ = 4.75, *p* < 0.001; Fig. 1, *A* and *C*). However, the level of pHDAC2^Ser394^ in the spinal cord did not differ significantly between the SNL and sham control group (n = 12 rats per group; Fig. 1, *B* and *D*). Furthermore, immunoblotting showed that SNL significantly increased the total HDAC2 protein levels (t_(22)_ = 3.49, *p* < 0.01) in the DRG but not in the spinal cord (n = 12 rats per group; Fig. 1, *A*–*D*). The pHDAC2/total HDAC2 ratio showed a significant increase in pHDAC2 levels relative to total HDAC2 in the injured DRG. (t_(22)_ = 3.40, *p* < 0.01, n = 12 rats per group; Fig. 1*E*). Nerve injury augments anterograde synaptic trafficking of α2δ-1 proteins from DRG neurons to their central terminals in the spinal dorsal horn (8). As expected, SNL also significantly increased the protein level of α2δ-1 (t_(22)_ = 5.10, *p* < 0.001) in the DRG as well as in the dorsal spinal cord (n = 12 rats per group; t_(22)_ = 7.28, *p* < 0.001; Fig. 1, *A*–*D*). These data indicate that nerve injury increases serine phosphorylation of HDAC2 with a concurrent increase in α2δ-1 expression in the DRG.

### Nerve injury augments the physical interaction between CK2 and HDAC2 in the DRG

**Figure 2:**
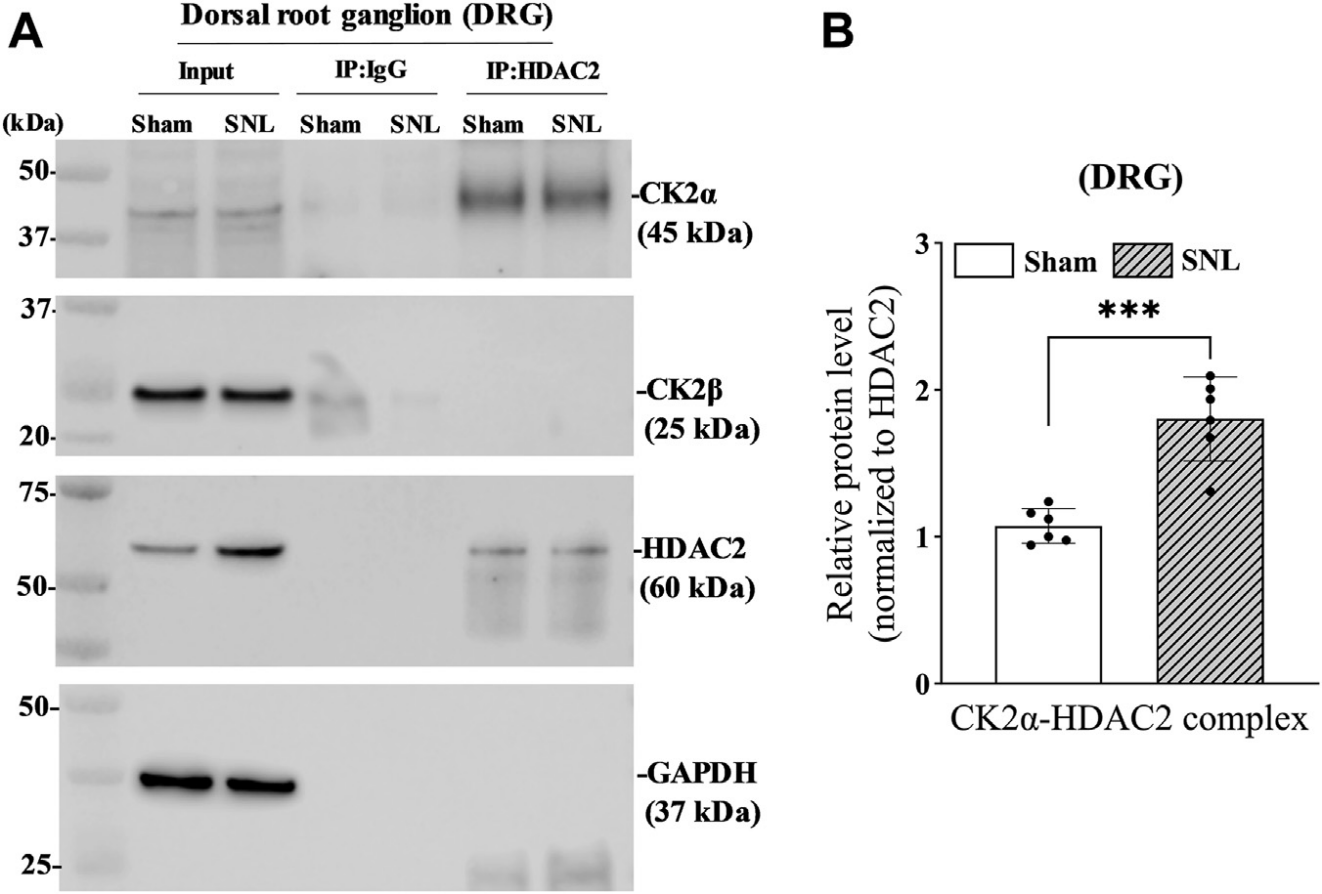
**Nerve injury augments the interaction between HDAC2 and CK2α in the DRG.***A*, representative immunoblotting images show that the anti-HDAC2 antibody precipitated CK2α, but not CK2β, in the DRG of rats subjected to spinal nerve ligation (SNL) or sham surgery. Three weeks after the surgery, total proteins from DRG tissues were extracted for immunoprecipitation (IP) with an anti-HDAC2 antibody. The immune complex was then subjected to immunoblotting for the detection of bound proteins. Normal rabbit IgG was used as a negative control in immunoprecipitation. *B*, quantification of the HDAC2-CK2α protein complex in the DRG from SNL and sham control rats (normalized to the HDAC2 protein band in IP samples, n = 6 rats per group). Data are expressed as means ± SD. ∗∗∗*p* < 0.001 (two-tailed Student *t* test).

CK2 is mainly associated with and phosphorylates HDAC2 in cancer cell lines (25). The Ser^394^ phosphorylation of HDAC2 is selectively catalyzed by CK2 (16, 17, 18). We next conducted co-immunoprecipitation assays to determine whether nerve injury affects the physical interaction between CK2 and HDAC2 in the DRG. DRG tissues at the L5 and L6 levels were removed from SNL and sham control rats 3 weeks after surgery. The anti-HDAC2 antibody precipitated CK2α, but not CK2β, proteins in DRG tissues in both SNL and sham groups (Fig. 2*A*). When normalized to the immunoprecipitated HDAC2 protein, the level of HDAC2-CK2α protein complexes was significantly greater in the SNL than in the sham control group (n = 6 rats per group; t_(10)_ = 3.45, *p* < 0.001; Fig. 2*B*). These results suggest that nerve injury enhances the direct interaction between HDAC2 and the catalytic CK2α subunit in the DRG.

### CK2 inhibition diminishes nerve injury-induced HDAC2 phosphorylation in the DRG

**Figure 3:**
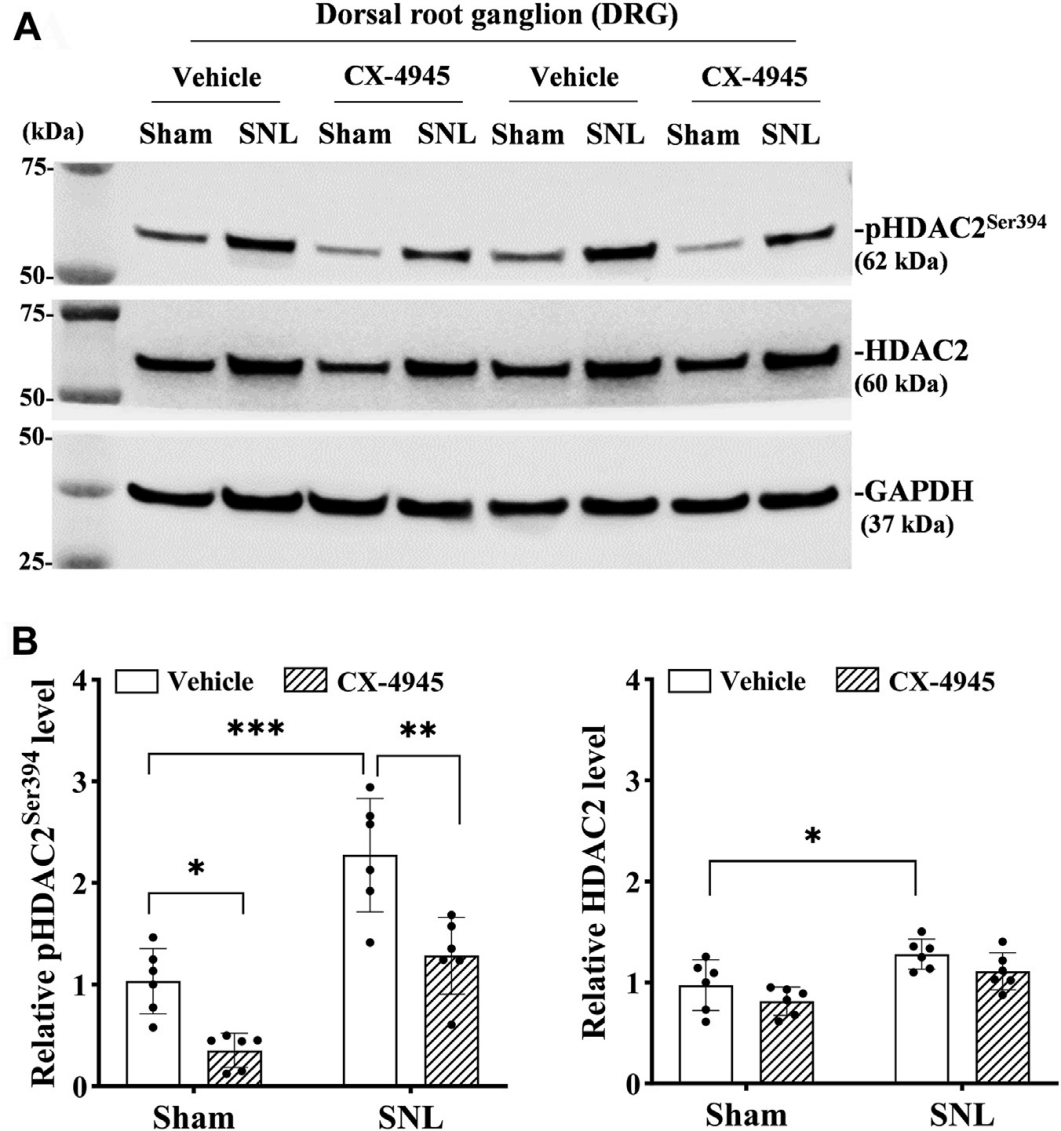
**CK2 inhibition attenuates nerve injury-induced HDAC2 phosphorylation in the DRG.***A* and *B*, Representative immunoblot images (*A*) and quantification (*B*) show the protein levels of phospho-serine^394^ of HDAC2 (pHDAC2^Ser394^) and total HDAC2 in the DRG of rats subjected to spinal nerve ligation (SNL) or sham surgery (n = 6 rats per group). SNL and sham control rats were intrathecally injected with vehicle or CX-4945 (10 μg per day) for five consecutive days. Total proteins were extracted 2 h after the last injection. GAPDH was used as a loading control. Two-way ANOVA showed that there was a significant main effect for SNL (F_(1,__20)_ = 36.9; *p* < 0.001) and CX-4945 treatment (F_(1,20)_ = 63.9; *p* < 0.001) and a significant interaction between SNL and CX-4945 treatment (F_(1,__20)_ = 5.19; *p* < 0.05). ∗*p* < 0.05, ∗∗*p* < 0.01, ∗∗∗*p* < 0.001 (two-way ANOVA followed by Tukey’s *post hoc* test). Data are expressed as means ± SD.

Because nerve injury potentiated HDAC2 serine phosphorylation and the CK2-HDAC2 interaction in the DRG, we then determined whether CK2 is solely responsible for the increased serine phosphorylation of HDAC2 by nerve injury. Three weeks after surgery, SNL and sham rats were treated with daily intrathecal injection of 10 μg CX-4945, a highly potent, ATP-competitive inhibitor of both isoforms of the CK2 catalytic subunits (α and α′) (26) or vehicle for five consecutive days. Immunoblotting showed that compared with the vehicle group, treatment with CX-4945 significantly inhibited Ser^394^ phosphorylation of HDAC2 in both sham (F_(1,20)_ = 63.9, *p* = 0.011) and SNL (F_(1,20)_ = 63.9, *p* = 0.003) rats (n = 6 rats per group; Fig. 3, *A* and *B*). However, treatment with CX-4945 had no significant effect on the total HDAC2 protein levels in the DRG from sham or SNL rats (n = 6 rats per group; Fig. 3*B*). These data indicate a critical role of nerve injury-inducing CK2 activity in the DRG in augmenting Ser^394^ phosphorylation of HDAC2. Two-way ANOVA showed a significant interaction between SNL and CX-4945 treatment on pHDAC2^Ser394^ levels in the DRG (F_(1,20)_ = 5.19, *p* < 0.05). These data indicate a critical role of CK2 activity in the phosphorylation of HDAC2 in the DRG augmented by nerve injury.

### CK2 inhibition attenuates pain hypersensitivity and α2δ-1 expression in the DRG induced by nerve injury

**Figure 4:**
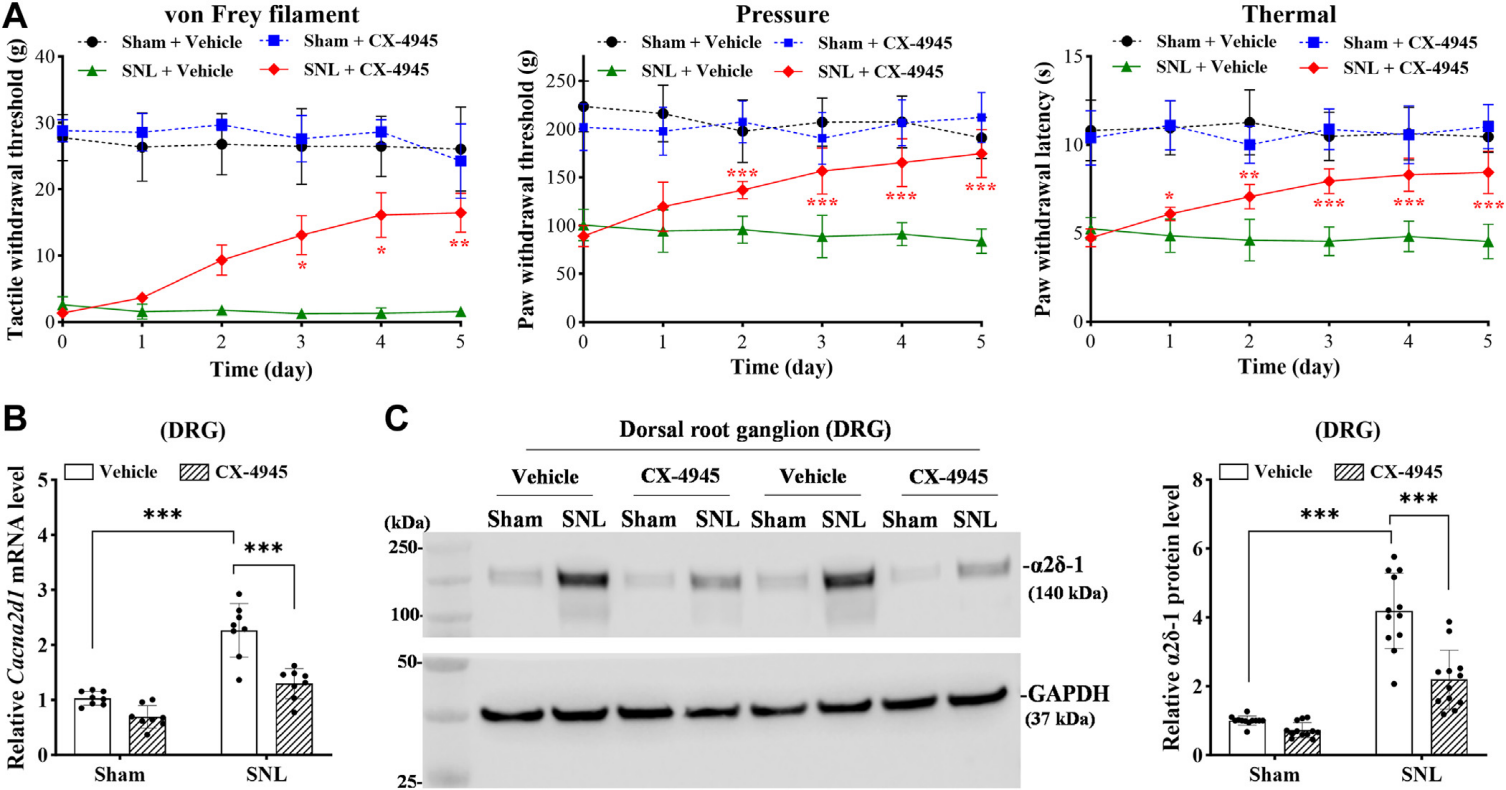
**CK2 inhibition attenuates nerve injury–induced pain hypersensitivity and α2δ-1 expression in the DRG.***A*, time course of changes in withdrawal thresholds tested with von Frey filaments, pressure, and thermal stimuli in sham and SNL rats intrathecally treated with vehicle or CX-4945 (10 μg per day) for five consecutive days (n = 8 rats per group). ∗*p* < 0.05, ∗∗*p* < 0.01, ∗∗∗*p* < 0.001, compared with SNL rats treated with vehicle at the same time point (two-way ANOVA with Tukey's *post hoc* test). *B*, quantitative PCR assays show the mRNA levels of *Cacna2d1* in the DRG of sham and SNL rats intrathecally injected with vehicle or CX-4945 (10 μg per day) for five consecutive days (n = 8 rats per group). Two-way ANOVA showed that there was a significant main effect for SNL (F_(1,__28)_ = 72.4; *p* < 0.001) and CX-4945 treatment (F_(1,28)_ = 36.6; *p* < 0.001) and a significant interaction between SNL and CX-4945 treatment (F_(1,__20)_ = 8.45; *p* < 0.01). ∗∗∗*p* < 0.001 (two-way ANOVA with Tukey's *post hoc* test). *C*, representative immunoblotting images and quantification show the protein levels of α2δ-1 in the DRG of sham and SNL rats intrathecally injected with vehicle or CX-4945 for five consecutive days (n = 12 rats per group). Total proteins were extracted 2 h after the last injection. GAPDH was used as a loading control. Two-way ANOVA showed that there was a significant main effect for SNL (F_(1,__44)_ = 130; *p* < 0.001) and CX-4945 treatment (F_(1,44)_ = 30.9; *p* < 0.001) and a significant interaction between the SNL and CX-4945 treatment (F_(1, 44)_ = 17.8; *p* < 0.001). ∗∗∗*p* < 0.001 (two-way ANOVA with Tukey's *post hoc* test). Data are expressed as means ± SD.

Intrathecal administration of CK2 inhibitors produces a prolonged inhibitory effect on pain hypersensitivity caused by nerve injury (22). To determine whether increased CK2 activity mediates nerve injury-induced increases in α2δ-1 expression in the DRG, we administered CX-4945 (10 μg per day) or vehicle *via* intrathecal injection 3 weeks after sham and SNL surgery. Throughout the course of the experiment, the baseline hind paw withdrawal thresholds in response to von Frey filaments (F_(15, 140)_ = 8.69), pressure (F_(15,__140)_ = 7.04), and thermal (F_(15,__140)_ = 4.13) stimuli were significantly (*p* < 0.001) lower in vehicle-treated SNL rats than in vehicle-treated sham rats (n = 8 rats per group; Fi. 4*A*). Daily intrathecal injection of 10 μg of CX-4945, but not the vehicle, for five consecutive days, led to a gradual increase in hind paw withdrawal thresholds in SNL rats (n = 8 rats per group; Fig. 4*A*). In contrast, intrathecal injection of CX-4945 had no significant effect on the hind paw withdrawal thresholds in sham control rats (n = 8 rats per group; Fig. 4*A*).

We next utilized quantitative PCR (qPCR) and immunoblotting to quantify mRNA and protein levels of α2δ-1, respectively. DRG tissues at the L5 and L6 levels were obtained from SNL and sham rats treated intrathecally with CX-4945 or vehicle for 5 days. In vehicle-treated rats, SNL caused a large increase in the transcript and protein levels of α2δ-1 in the DRG. Treatment with CX-4945 profoundly reduced the mRNA level of α2δ-1 in the DRG of SNL rats (n = 8 rats per group, F_(1,28)_ = 36.6, *p* < 0.001; Fig. 4*B*). Also, treatment with CX-4945 diminished the SNL-induced high protein level of α2δ-1 in the DRG (n = 12 rats per group, F_(1,__44)_ = 30.9, *p* < 0.001; Fig. 4*C*). These findings suggest that elevated CK2 activity by nerve injury plays a key role in α2δ-1 upregulation in the DRG.

### CK2 inhibition restores HDAC2 enrichment at the *Cacna2d1* promoter in the injured DRG

**Figure 5:**
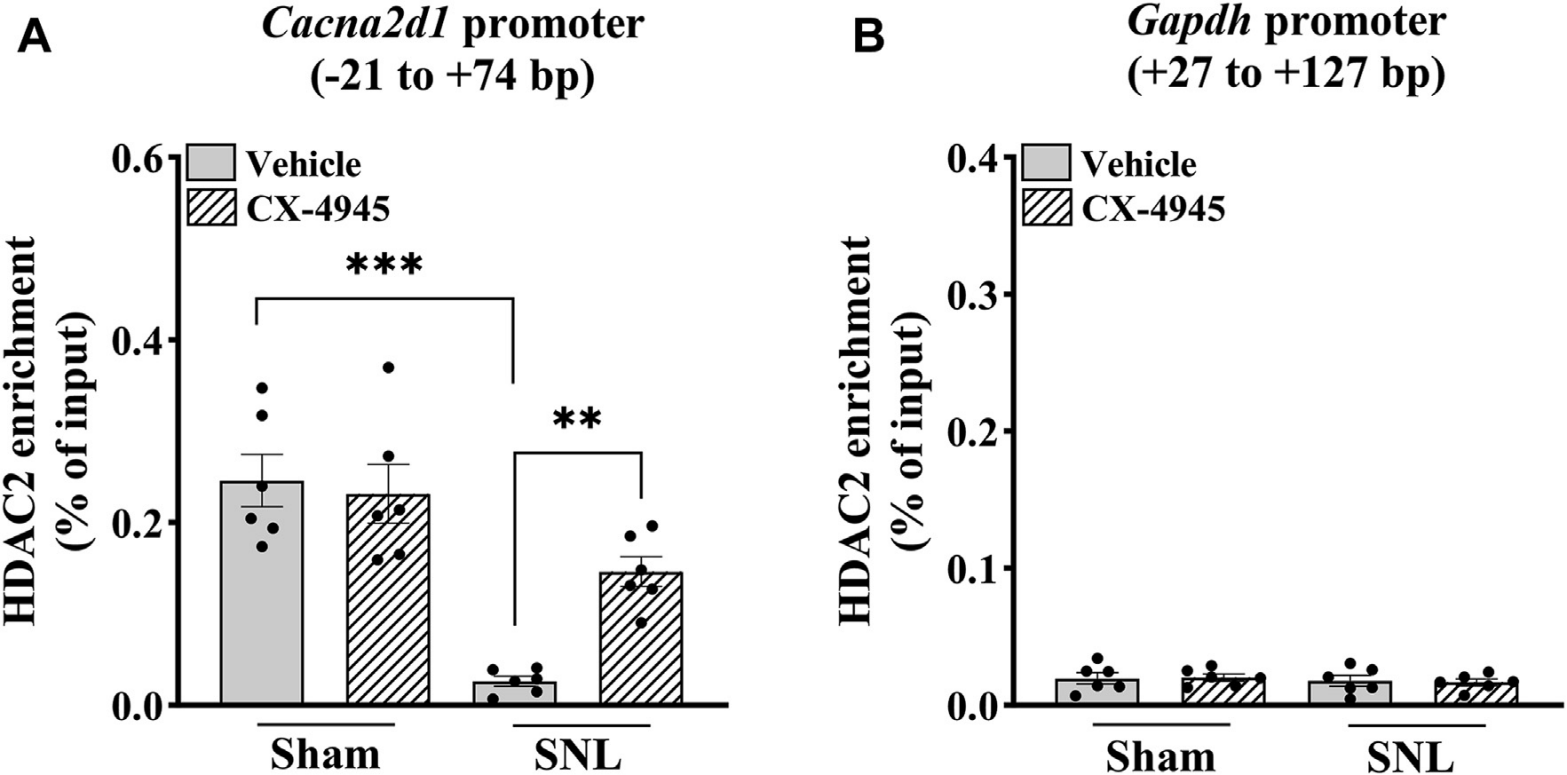
**CK2 inhibition restores HDAC2 enrichment at the *Cacna2d1* promoter in the injured DRG.***A* and *B*, quantification of the immunoprecipitated chromatin show the enrichment of HDAC2 proteins at the −21 to +74 bp region of the *Cacna2d1* promoter (*A*) and in the +27 to +127 bp region of the *Gapdh* promoter (*B*) in the DRG. ChIP-qPCR was performed using the DRG tissues from sham and SNL rats intrathecally injected with vehicle or CX-4945 (10 μg per day) for five consecutive days (n = 6 rats per group). Two-way ANOVA showed that there was a significant main effect for SNL (F_(1, 20)_ = 42.9; *p* < 0.001) and CX-4945 treatment (F_(1,20)_ = 5.17; *p* < 0.01) and a significant interaction between SNL and CX-4945 treatment (F_(1,__20)_ = 8.42; *p* < 0.01). ∗∗*p* < 0.01, ∗∗∗*p* < 0.001 (two-way ANOVA with Tukey's *post hoc* test). Data are expressed as means ± SD.

Nerve injury potentiates α2δ-1 expression *via* inducing HDAC2 dissociation from the *Cacna2d1* promoter region in the DRG (11). Next, we used chromatic immunoprecipitation (ChIP)-qPCR to determine the role of CK2 activity in the control of HDAC2 occupancy at the *Cacna2d1* gene promoter in the injured DRG. We obtained DRG tissues at the L5 and L6 levels from SNL and sham rats treated intrathecally with CX-4945 (10 μg per day) or vehicle for 5 days. Similar to what we reported previously (11), the amount of HDAC2 at the −21 to +74 base pair (bp) region across the transcription start site of *Cacna2d1* in the DRG was much lower in vehicle-treated SNL rats than in vehicle-treated sham control rats (n = 6 rats per group, F_(1,20)_ = 42.9; *p* < 0.001; Fig. 5*A*). Treatment with CX-4945 largely restored HDAC2 enrichment in the same *Cacna2d1* promoter region in the injured DRG (n = 6 rats per group; Fig. 5*A*). SNL or CX-4945 had no significant effect on the HDAC2 enrichment at the −27 to +127 bp region of the *Gapdh* promoter in the DRG (n = 6 rats per group; Fig. 5*B*). These results suggest that increased CK2 activity plays a major role in nerve injury-induced HDAC2 dissociation from the *Cacna2d1* promoter region in the DRG.

### CK2 inhibition reduces histone acetylation and active histone marks at the *Cacna2d1* promoter in the injured DRG

**Figure 6:**
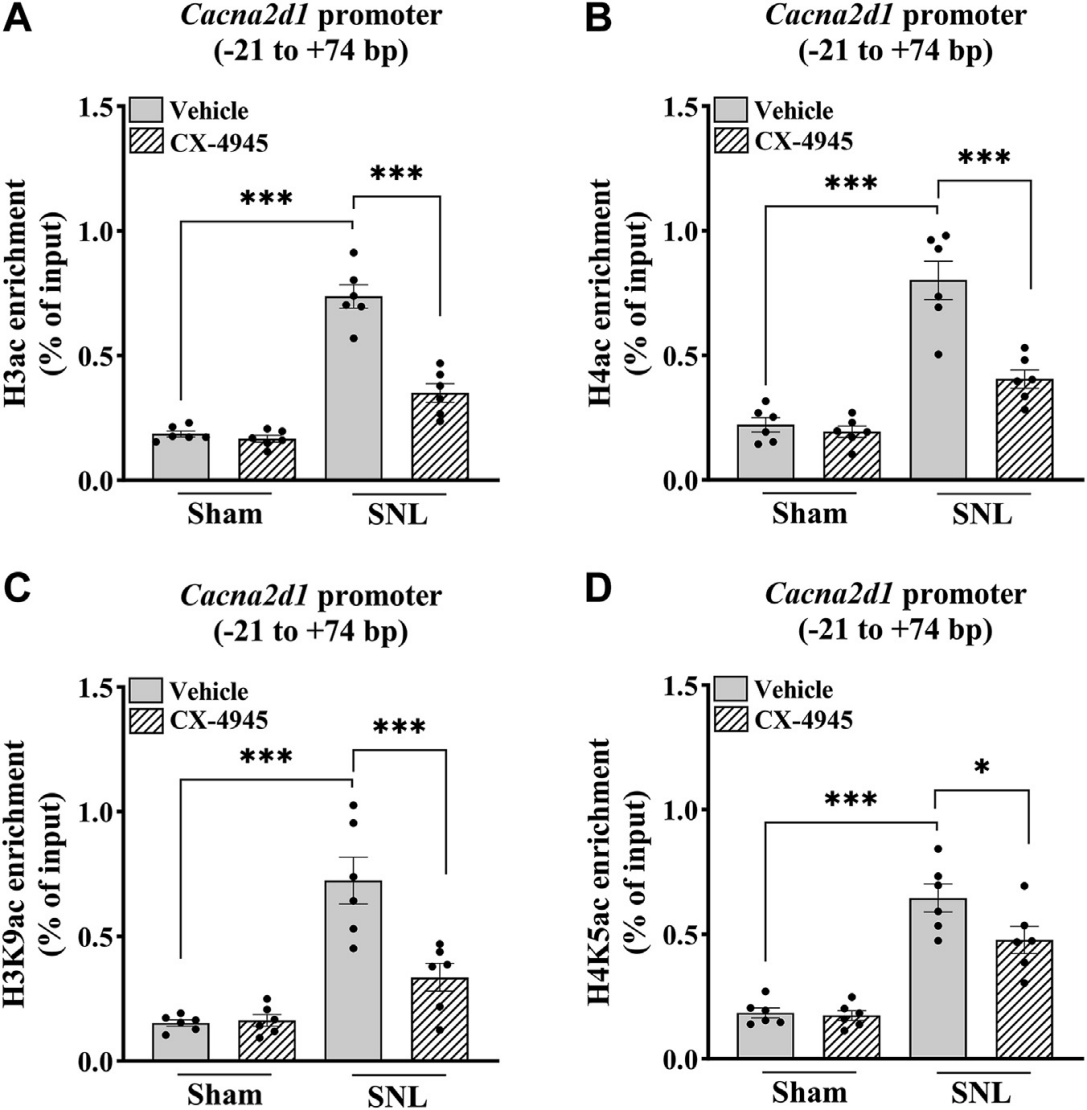
**CK2 inhibition reduces histone acetylation at the *Cacna2d1* promoter in the injured DRG.***A*–*D*, quantification of the immunoprecipitated chromatin show the enrichment of total H3ac (*A*), H4ac (*B*), H3K9ac (*C*), and H4K5ac (*D*) at the *Cacna2d1* promoter (−21 to +74 bp). ChIP-qPCR was performed using the DRG tissues from sham and SNL rats intrathecally injected with vehicle or CX-4945 (10 μg per day) for five consecutive days (n = 6 rats per group). Two-way ANOVA showed that there was a significant interaction between SNL and CX-4945 treatment for the enrichment of H3ac (F_(1,__20)_ = 34.4; *p* < 0.001), H4ac (F_(1,__20)_ = 15.7; *p* < 0.001), and H3K9ac (F_(1,__20)_ = 12.7; *p* < 0.01). ∗*p* < 0.05, ∗∗∗*p* < 0.001 (two-way ANOVA with Tukey's *post hoc* test). Data are expressed as means ± SD.

Because the abundance of HDAC2 at the *Cacna2d1* promoter actively controls histone acetylation levels in the DRG (11), we determined whether CK2 activity controls the level of acetylated H3 and H4 histones at the *Cacna2d1* promoter in the injured DRG. DRG tissues at the L5 and L6 levels were harvested from SNL and sham rats treated intrathecally with CX-4945 (10 μg per day) or vehicle for 5 days. ChIP-qPCR assays showed that the total H3ac (F_(1,__20)_ = 138; *p* < 0.001) and H4ac (F_(1,__20)_ = 72.3; *p* < 0.001) levels at the −21 to +74 bp region of the *Cacna2d1* promoter in the DRG were much greater in vehicle-treated SNL rats than in vehicle-treated sham rats (n = 6 rats group; Fig. 6, *A* and *B*). Treatment with CX-4945 reversed SNL-induced increases in total H3ac and H4ac levels at the *Cacna2d1* promoter in the DRG (n = 6 rats group; Fig. 6, *A* and *B*).

Finally, because H3K9ac and H4K5ac are well-known histone marks associated with active gene transcription (11, 12, 27, 28), we also determined whether CK2 activity regulates the enrichment of H3K9ac and H4K5ac at the −21 to +74 bp region of the *Cacna2d1* promoter in the injured DRG. ChIP-qPCR assays showed that H3K9ac (F_(1,__20)_ = 44.2; *p* < 0.001) and H4K5ac (F_(1,__20)_ = 85.3; *p* < 0.001) levels at the *Cacna2d1* promoter in the DRG were significantly higher in vehicle-treated SNL rats than in vehicle-treated sham rats (n = 6 rats group; Fig. 6, *C* and *D*). These SNL-induced increases in H3K9ac and H4K5ac enrichment at the *Cacna2d1* promoter in the DRG were largely attenuated by intrathecal treatment with CX-4945 (n = 6 rats group; Fig. 6, *C* and *D*). Together, these findings suggest a significant role of CK2 activity and the CK2-HDAC2 interaction in the control of nerve injury-induced potentiation of histone acetylation and active histone marks at the *Cacna2d1* promoter in the DRG.

## Discussion

Our study provides new evidence that increased CK2 activity plays a key role in nerve injury-induced α2δ-1 expression in the DRG. Upregulation of α2δ-1 in the DRG is closely associated with the development of chronic neuropathic pain caused by traumatic nerve injury and chemotherapy (6, 7, 8, 9). The increased availability of α2δ-1 proteins in DRG neurons can form a complex directly with phosphorylated NMDARs to promote synaptic trafficking and activity of NMDARs at the central terminals of DRG neurons in the spinal cord (3, 8, 9, 29). This increased presynaptic NMDAR activity enhances glutamate release to excitatory neurons in the spinal dorsal horn, leading to increased excitation of postsynaptic neurons and amplification of nociceptive transmission (8, 10, 30). This mechanism of action accounts for the therapeutic effect of gabapentin and pregabalin, inhibitory ligands of α2δ-1 proteins, in neuropathic pain conditions (3, 8, 9, 10). We have shown previously that inhibition of CK2 with 5,6dichlorobenzimidazone-1-β-D-ribofuranoside (DRB) or 4,5,6,7-tetrabromobenzotriazole (TBB) diminishes synaptic NMDAR hyperactivity and pain hypersensitivity caused by nerve injury or calcineurin inhibitors (22, 23, 31). NMDARs may be a direct substrate of CK2 (22, 32), and inhibiting CK2 activity could attenuate synaptic NMDAR activity by diminishing NMDAR phosphorylation potentiated by nerve injury. In the present study, we demonstrated that the potent and specific CK2 inhibitor CX-4945 reversed pain hypersensitivity caused by nerve injury but had no effect on normal nociception in control rats. Importantly, CK2 inhibition largely normalized α2δ-1 expression levels in the injured DRG without affecting α2δ-1 expression in the control DRG. These findings suggest that nerve injury enhances CK2 activity in the DRG, which in turn facilitates α2δ-1 upregulation and pain hypersensitivity. Thus, increased CK2 activity in the DRG likely has a dual role in augmented NMDAR activity in neuropathic pain (1), enhancing NMDAR phosphorylation and (2) potentiating α2δ-1 expression.

A major finding of our study is that nerve injury increases the CK2-HDAC2 interaction and CK2-mediated HDAC2 phosphorylation in the DRG. Epigenetic mechanisms, which involve reversible modifications to chromatin DNA and histones, play pivotal roles in the transcriptional regulation of genes engaged in neuropathic pain development (12, 33, 34, 35). HDAC2 is the most abundant histone deacetylase expressed in DRG neurons (11, 12). HDAC2 removes acetyl groups from histone proteins, leading to chromatin remodeling and gene transcriptional repression. Conditional HDAC2 knockout in DRG neurons causes α2δ-1 upregulation and neuropathic pain-like phenotype in mice (11), indicating a pivotal role of HDAC2 in constitutively restraining α2δ-1 expression in DRG neurons. Structurally, HDAC2 comprises the N-terminal histone deacetylase catalytic domain (9–322) and a flexible C-terminus with a characteristic coiled-coil domain responsible for the regulation of enzyme activity (36). Serine phosphorylation of HDACs acts as a regulatory mechanism of their deacetylase activity and epigenetic control of gene transcription. For example, CK2-mediated phosphorylation of Ser^421^ and Ser^423^ residues of HDAC1 is highly correlated with its enzymatic activity and interaction with other proteins in forming complexes (37). Unlike HDAC1, which is phosphorylated by CK2, cAMP-dependent protein kinase, and protein kinase G, HDAC2 phosphorylation at Ser^394^, Ser^422^, and Ser^423^ is catalyzed uniquely by CK2 (16, 17, 18, 38, 39). In this study, we found that nerve injury-induced a large increase in phosphorylation of HDAC2 at Ser^394^ in the DRG. Remarkably, CK2 inhibition with CX-4945 largely attenuated the phosphorylation level of HDAC2 in the injured DRG, suggesting a crucial role of CK2 in nerve injury-induced HDAC2 phosphorylation. We observed that treatment with CX-4945 did not fully restore phospho-HDAC2 levels in the injured DRG to those observed in sham DRG, indicating that protein kinases other than CK2 may also contribute to nerve injury-induced HDAC2 phosphorylation. Alternatively, the dose of CX-4945 used in our study may not have fully inhibited CK2 activity. CK2 is a constitutively active Ser/Thr protein kinase composed of two catalytic (α or α′) subunits and two regulatory β subunits (19). We showed that CK2α, the catalytic subunit of CK2, but not CK2β, physically interacted with HDAC2 in the DRG, and this interaction was significantly potentiated by nerve injury. Thus, CK2α may directly catalyze the HDAC2 phosphorylation involved in controlling the deacetylase activity of HDAC2 in the DRG.

Notably, the CK2α-HDAC2 interaction is also present in the control DRG, indicating a possible dynamic balance between the phosphorylated HDAC2 and unphosphorylated HDAC2 regulated by CK2 activity under normal conditions. We found that treatment with CX-4945 decreased pHDAC2 levels in the DRG of sham animals, suggesting that HDAC2 phosphorylation is constitutively controlled by CK2. HDAC2 is assembled into the core component of several multiprotein complexes, such as the nuclear remodeling and deacetylation (NuRD), switch-independent 3 (SIN3), co-repressor for element-1-silencing transcription factor (CoREST), and mitotic deacetylase (MiDAC), that remodel chromatin and control gene transcription (40, 41, 42, 43, 44). Together with other interacting proteins, CK2-mediated signaling likely plays a crucial role in regulating α2δ-1 expression in the injured DRG. It remains unclear whether CK2α-mediated HDAC2 phosphorylation alters its interactions with its partners in the corepressor complexes or other transcription factors such as Sp3, KLF4, and FOXO3a (27, 28, 45, 46). The transcriptional repressor Sp3 is actively associated with CK2-phosphorylated HDAC2 in cell lines (25). Thus, the CK2α-mediated phosphorylation of HDAC2 could potentially regulate its protein-protein interactions, corepressor organization, and functional activity (15, 38). Also, the multi-subcellular localization of CK2 depends on the physiological conditions (47). Further studies are needed to determine whether nerve injury changes CK2 colocalization in the nucleus or other compartments in DRG neurons.

Another significant finding of our study is that increased CK2 activity plays a crucial role in nerve injury-induced HDAC2 dissociation and histone hyperacetylation at the *Cacna2d1* promoter in the DRG. Under normal conditions, HDAC2 constitutively binds to the *Cacna2d1* promoter, maintaining low histone acetylation levels and limiting *Cacna2d1* transcription in the DRG. We have shown that nerve injury diminishes HDAC2 occupancy in the region from −32 to +60 bp at the *Cacna2d1* promoter in the DRG, promoting histone hyperacetylation in this region (11). The removal of HDAC2 from the *Cacna2d1* promoter could expose the critical lysine residues on histones H3 and H4 for enhanced acetylation, shifting from transcriptional repression to activation. In this study, we found a similar loss of HDAC2 from the −21 to +74 bp region spanning the transcription start site in the injured DRG. Remarkably, we found that CK2 inhibition with CX-4945 largely restored HDAC2 enrichment at the *Cacna2d1* promoter in the injured DRG but had no such effect in the control DRG. These results suggest that nerve injury–enhanced CK2 activity plays a key role in diminishing HDAC2’s binding capacity (either individually or within its corepressor complexes) at the *Cacna2d1* promoter in the DRG. Additionally, we observed that CK2 inhibition decreased acetylation levels of histones H3 and H4, particularly H3K9ac and H4K5ac (two well-known histone activating marks) (11, 35), at the *Cacna2d1* promoter in the injured DRG. Thus, CK2 can dynamically control the abundance and repressive activity of HDAC2 at the *Cacna2d1* promoter by regulating the phosphorylation status of HDAC2 in the DRG. Our findings support the notion that CK2-regulated HDAC2 phosphorylation is a critical molecular mechanism determining HDAC2 enrichment at the *Cacna2d1* promoter and enhanced *Cacna2d1* transcription in the DRG in neuropathic pain. We acknowledge that CK2 activity likely controls other nociceptive genes and the recruitment of transcription factors and histone modifiers other than HDAC2 in the DRG. Further studies are needed to unravel whether other nociceptive genes, transcription factors (*e.g.*, Sp3 and Co-REST), and histone enzymes (*e.g.*, histone acetyltransferases) involved in histone acetylation are controlled by CK2 in the development of neuropathic pain.

**Figure 7:**
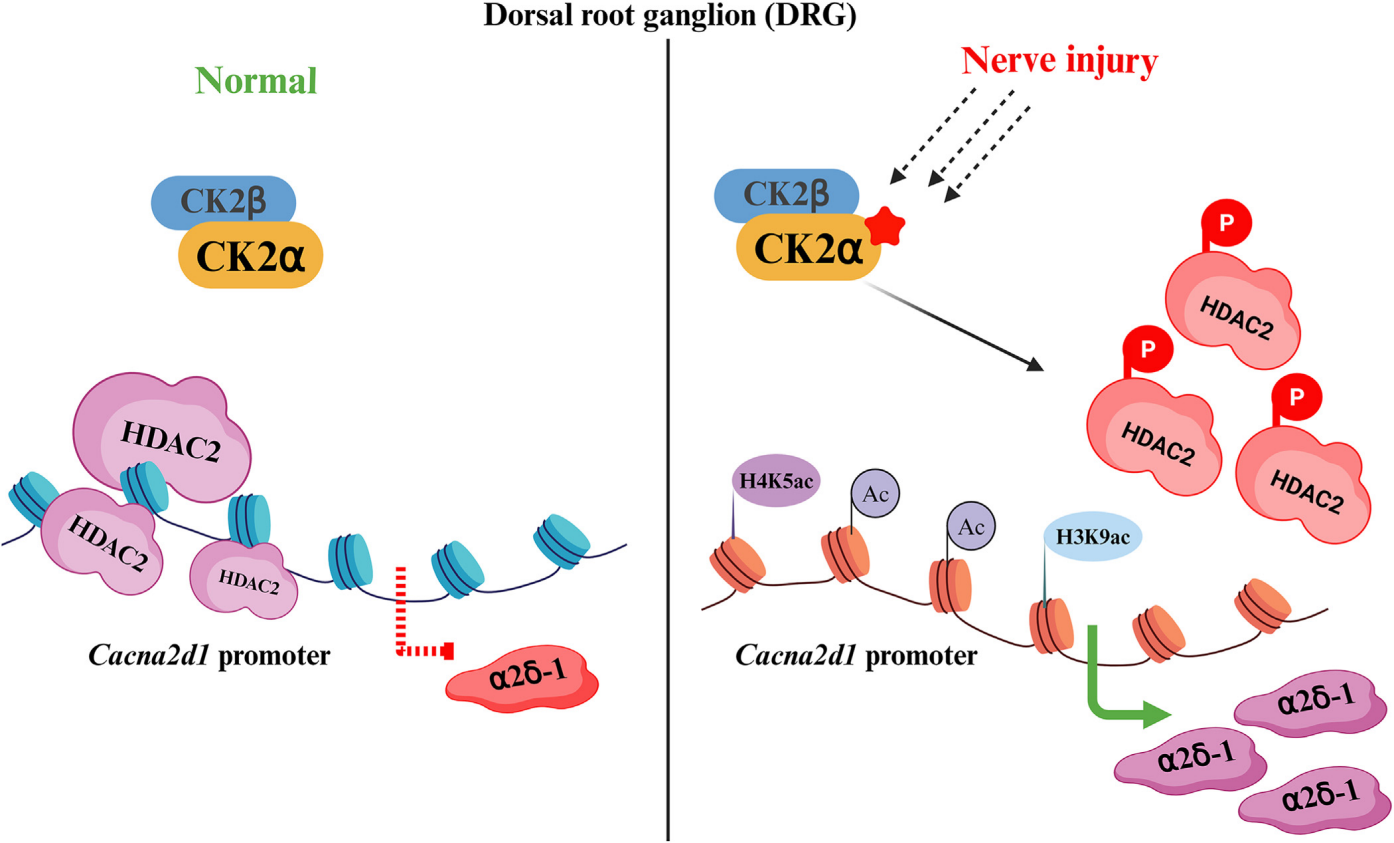
**Schematic representation illustrates changes induced by nerve injury in CK2 and HDAC2 phosphorylation, histone acetylation at the *Cacna2d1* promoter, and α2δ-1 expression in the DRG.** Under normal conditions, CK2 minimally interacts with HDAC2, which is highly enriched by the *Cacna2d1* gene promoter. Consequently, the acetylation levels of histones H3 and H4, alone with activating histone marks (H3K9ac or H4K5ac), are low at the *Cacna2d1* promoter, keeping the *Cacna2d1* transcription epigenetically repressed in the DRG. Nerve injury augments CK2 activity, leading to increased CK2-HDAC2 interactions, HDAC2 phosphorylation, and subsequent HDAC2 dissociation at the *Cacna2d1* promoter in the DRG. The loss of HDAC2 at the *Cacna2d1* promoter boosts histone acetylation levels and the presence of active histone marks, resulting in elevated *Cacna2d1* transcription and α2δ-1 expression in the injured DRG.

In summary, our study provides new evidence about epigenetic mechanisms regulating *Cacna2d1* transcription in the DRG in neuropathic pain. Our findings suggest that traumatic nerve injury increases CK2 activity and its interaction with HDAC2. Subsequent HDAC2 phosphorylation reduces HDAC2 occupancy and elevated histone acetylation at the *Cacna2d1* promoter, rendering it transcriptionally active (Fig. 7). These new findings explain how nerve injury induces HDAC2 detachment from the *Cacna2d1* promoter, promoting *Cacna2d1* transcription in primary sensory neurons in neuropathic pain. Because α2δ-1 proteins are essential for the heightened activity of synaptic NMDARs and calcium permeable-AMPA receptors (8, 10, 48), CK2 activity, HDAC2 phosphorylation, and HDAC2-controlled histone acetylation at the *Cacna2d1* promoter are mutually involved in nerve injury-induced synaptic plasticity and amplification of nociceptive signals. Our findings further suggest that CK2 is a promising target for treating chronic neuropathic pain by restoring the repressive function of HDAC2 and restraining *Cacna2d1* transcription in primary sensory neurons.

## Experimental procedures

### Rat model of neuropathic pain

Male Sprague-Dawley rats (8–10 weeks old) were obtained from Envigo (Indianapolis, IN). All surgical procedures and experimental protocols were approved by the University of Texas MD Anderson Cancer Center Animal Care and Use Committee and adhered to the National Institutes of Health guidelines on the ethical use of animals. Spinal nerve ligation (SNL) was used as an experimental model of neuropathic pain. In brief, anesthesia was induced using 2 to 3% isoflurane, the left L5 and L6 spinal nerves were then isolated under a surgical microscope and ligated using a 5-0 silk suture (22, 49, 50). Control animals underwent a sham surgical procedure without nerve ligation. In the SNL model, stable pain hypersensitivity typically develops 10 to 14 days after surgery and persists for at least 8 weeks (4, 22).

### Intrathecal catheterization and treatment

Two weeks after surgery, intrathecal catheters were implanted in selected SNL and sham-operated rats under isoflurane-induced anesthesia. First, a small incision was made at the back of the animal's neck. Subsequently, a small opening was created in the atlanto-occipital membrane of the cisterna magna, and a PE-10 catheter was inserted. The catheter was carefully positioned so that its caudal tip reached the lumbar spinal cord (22, 51). The rostral end of the catheter was externalized, and the wound was closed using sutures. Following the surgery, the animals were given 5 to 7 days to recover. Rats exhibiting motor or neurological impairment after catheter insertion were promptly euthanized. The selective CK2 inhibitor, CX-4945 (#HY-50855; MedChemExpress, Monmouth Junction, NJ) or a vehicle consisting of 10% dimethyl sulfoxide and 90% saline mixed with sulfobutylether-β-cyclodextrin (#HY-17031, MedChemExpress) was administered intrathecally at a volume of 10 μl, followed by a 5 μl saline flush.

### Nociceptive behavioral tests

Nociceptive behavioral assays were conducted in rats 2 to 3 weeks after surgery as described previously (11, 22). To measure tactile sensitivity, rats were allowed to acclimate on a wire-grid floor for 30 min prior to testing. von Frey filaments (Stoelting) of varying forces were applied to the plantar surface of the hind paw with sufficient pressure to bend the filament for 6 s. A brisk withdrawal or paw flinching was recorded as a positive response. If no response was observed, the next filament with greater force was applied. The "up-down" method was utilized to determine the 50% likelihood of a withdrawal response (51, 52).

For the assessment of mechanical nociception, a paw pressure test was conducted using an analgesiometer (Ugo Basile). The test involved pressing a foot pedal to activate a motor, which applied a linearly increasing force to the dorsal surface of the hind paw. The pedal was released immediately upon the animal displaying signs of pain, such as paw withdrawal or vocalization. Each trial was repeated two or three times at 2-min intervals, and the average force required to elicit a withdrawal response was calculated (53, 54). A maximum force of 400*g* was set as a cutoff to prevent potential tissue injury to the animals.

The heat sensitivity of the hindpaw was evaluated using a Plantar Analgesia Meter (model 400, IITC Life Science). The plantar surface was maintained at 30 °C, and animals were acclimatized for 30 min before testing. A mobile radiant heat stimulus was directed onto the plantar surface of the hind paw until the animal exhibited a withdrawal response or began paw licking (55). A cutoff time of 30 s was implemented to reduce the risk of potential tissue injury.

### Coimmunoprecipitation

For coimmunoprecipitation, total proteins were extracted from the DRG or dorsal spinal cord tissues using Pierce IP lysis buffer (#87788, Thermo Scientific) supplemented with a Halt protease and phosphatase inhibitor cocktail (#78442, Thermo Scientific). Equal amounts (1 mg) of total proteins from both sham and SNL groups were incubated at 4 °C overnight with protein G agarose fast flow beads (#16–266, Millipore Sigma) and 2 μg of rabbit anti-HDAC2 antibody (#GTX109642, GeneTex) or normal rabbit IgG (#2729S, Cell Signaling Technology, Danvers, MA). Following incubation, all samples were washed three times with Pierce IP lysis. The beads were then incubated with 1 × NuPAGE LDS sample buffer (#NP0007, Invitrogen) along with 1 × NuPAGE sample reducing agent (#NP0009, Invitrogen) for 10 min at 25 °C with rotation, followed by heating for 5 min at 70 °C. The eluted proteins were subjected to further immunoblotting analysis. The protein bands of CK2α and CK2β were normalized to those of HDAC2 on the same blots.

### Immunoblotting

For immunoblotting, the flash-frozen tissues from sham and SNL rats were homogenized in ice-cold radioimmunoprecipitation (RIPA) lysis and extraction buffer (#89900; Thermo Scientific) supplemented with 1 × Halt protease and phosphatase inhibitor cocktail. The concentration of the extracted total protein was determined using the DC protein assay kit (Bio-Rad Laboratories) with bovine serum albumin (BSA) as a reference standard. Equal amounts of proteins extracted or eluted from coimmunoprecipitation from all samples were subjected to reducing and denaturing gel electrophoretic separation on NuPAGE 4 to 12% Bis-Tris mini protein gels (1.5 mm, #NP0335BOX, Invitrogen). Subsequently, the proteins on the gel were transferred to a 0.2 μm polyvinylidene difluoride (PVDF) membrane using a Trans-Blot turbo transfer system (Bio-Rad) for 10 min at a constant current of 2.5 A and up to 25 V. The membrane was immediately submerged in SuperBlock T20 (TBS) blocking buffer (#37536, Thermo Scientific) and blocked for 10 min at 25 °C under constant agitation. The membrane was then incubated overnight at 4 °C with the following primary antibodies diluted in the blocking buffer: mouse anti-HDAC2 (1:1000, #5113S, Cell Signaling Technology), rabbit anti-phospho-HDAC2 (Ser^394^) (1:1000, #69238S, Cell Signaling Technology), mouse anti-α2δ-1 (1:1000, #SC-271697, Santa Cruz Biotechnology), mouse anti-CK2α (1:500, #SC-12738, Santa Cruz Biotechnology), mouse anti-CK2β (1:500, #SC-46666, Santa Cruz Biotechnology), and rabbit anti-GAPDH (1:1000, #5174S, Cell Signaling Technology). The specificity of the anti-α2δ-1 antibody was validated using α2δ-1 knockout mice (29), whereas the specificity of anti-HDAC2, anti-CK2α, and anti-CK2β antibodies was confirmed by knockdown using siRNA/shRNA (22, 56, 57, 58). Subsequently, a goat anti-rabbit horseradish peroxidase-linked antibody (1:5000, #7074S, Cell Signaling Technology) or anti-mouse horseradish peroxidase-linked antibody (1:5000, #7076S, Cell Signaling Technology) was applied to the immunoblots for 2 h at 25 °C with constant agitation. To detect protein expression levels from coimmunoprecipitation samples, HRP-conjugated Trueblot secondary antibodies were used (1:5000, anti-rabbit IgG, #18-8816-31; anti-mouse IgG, #18-8817-31, Rockland Immunochemicals). The membranes were thoroughly washed three times after each antibody incubation. The protein bands on the membrane were visualized using the SuperSignal West Femto Maximum Sensitivity Substrate (#34094, Thermo Fisher Scientific) in the Odyssey Fc Imager (LI-COR Biosciences). The protein bands were then normalized to the corresponding glyceraldehyde-3-phosphate dehydrogenase (GAPDH) protein band on the same blot. ImageJ (https://imagej.net/ij/) software was used for the quantification of the protein bands. Empiria Studio software (Version 3.2; LI-COR Biosciences) was used for the analysis of the protein band intensity. The amount of target proteins in each sample was first normalized to the level of GAPDH, then to its expression level in sham or vehicle-treated rats. The mean value in the sham or vehicle control group was set to 1.

### Quantitative polymerase chain reaction (qPCR)

Total RNAs were extracted from the lumbar DRG and dorsal spinal cord tissues using the RNeasy Plus Universal Mini Kit (#73404, Qiagen, Hilden, Germany). Following RNA extraction, equal amounts of RNA were treated with DNase to remove DNA contamination and subjected to reverse transcription to generate complementary DNA (cDNA) using the SuperScript IV VILO Master Mix with ezDNase Enzyme (#11766050, Invitrogen). For quantitative PCR (qPCR) analysis, 10 ng (2 μl) of diluted cDNA was utilized in a 20 μl reaction volume (in triplicates). This cDNA was mixed with PowerUp SYBR Green Master Mix (#A25776, Applied Biosystems, Waltham, MA) along with primers specific for rat *Cacna2d1* (forward: 5′-GGA CCT ATT CAG TGG ATG GCT TG-3′; reverse: 5′-CCA TTG GTC TTC CCA GAA CAT CTA GA-3′) and *Gapdh* (forward: 5′-CAT CCC AGA GCT GAA CGG GAA G-3′; reverse: 5′-GTC CTC AGT GTA GCC CAG GAT GC-3′) messenger RNA (mRNA). The QuantStudio 7 Flex Real-Time PCR System (Applied Biosystems) was employed for qPCR analysis using the following thermal cycling conditions: 50 °C for 2 min, 95 °C for 2 min, followed by 40 cycles of 95 °C for 5 s, and 60 °C for 30 s. To ensure specificity, the qPCR products were confirmed using melting curve analysis and agarose gel electrophoresis. Relative mRNA levels were determined using the 2^-ΔΔC^_T_ method (59), first normalized to the level of *Gapdh* mRNA in the same sample, and then to its level in sham or vehicle-treated rats. The mean value in the sham or vehicle control group was set to 1.

### Chromatin immunoprecipitation (ChIP)-qPCR

Chromatin immunoprecipitation assay was performed with some modifications to the previously described method (11, 60). The truChIP Chromatin Shearing Tissue Kit with formaldehyde (#520237, Covaris) was used to shear chromatin from the DRG tissues of sham and SNL rats. In brief, L5 and L6 DRGs (pooled from two rats for each sample) were washed with ice-cold PBS (phosphate-buffered saline solution, pH 7.2) and incubated in the fixing buffer with 2% formaldehyde (methanol free) for 10 min at 25 °C. After quenching the cross-linking reaction by Quenching the buffer for 5 min at 25 °C, the fixed tissue was washed three times with cold PBS. The fixed DRGs were incubated in lysis buffer B containing protease inhibitor for 20 min on a rotator at 4 °C with 3 s vertexing after 10 min. The nuclei were pelleted by centrifugation at 2000*g* for 5 min at 4 °C followed by incubation with wash buffer C containing protease inhibitor for 10 min on a rotator at 4 °C. The nuclear pellet was washed again and resuspended in 130 μl shearing buffer D2 (1 mM EDTA, 10 mM Tris-HCl at pH 7.6, and 0.25% SDS) containing the protease inhibitor. After a 10-min incubation on ice with frequent vertexing, the chromatin was transferred to a microTUBE–130 and sonicated with an adaptive focused acoustics ultrasonicator (#M220, Covaris) for 8 min at 4 °C with operating conditions including 75W power, 5% duty factor, and 200 cycles per burst (CPB) to achieve 200 to 700 bp chromatin fragments. The sized chromatin is diluted in 3X IP dilution buffer in 1:2 ratio and centrifuged at 10,000*g* for 5 min at 4 °C to obtain a clear supernatant for immunoprecipitation (IP). A 10% (volume) of the diluted chromatin was stored at −20 °C for extracting input DNA. A 5 μg of diluted chromatin was incubated overnight at 4 °C with rotation by adding following ChIP-validated antibodies (11, 12, 61): rabbit IgG (#2729S, Cell Signaling Technology), rabbit HDAC2 (#GTX109642, GeneTex), rabbit H3ac (#06–599, Millipore Sigma), rabbit H4ac (#06–866, Millipore Sigma), rabbit H3K9ac (#9649S, Cell Signaling Technology), and rabbit H4K5ac (#8647, Cell Signaling Technology).

Following immunoprecipitation, 50 μl ChIP-grade protein G magnetic beads (#9006S, Cell Signaling Technology) were added to each immunoprecipitation reaction tube and incubated for 2 h at 4 °C with rotation. Subsequently, the beads were washed twice with a low salt buffer followed by another wash with a high salt buffer. The bound chromatin was eluted using ChIP elution buffer for 2 h at 65 °C in a thermomixer. The eluted chromatin was de-crosslinked with 5 M NaCl and Proteinase K treatment for 8 h at 65 °C in a thermomixer followed by the purification of DNA using a QIAquick PCR Purification Kit (#28104, Qiagen). The purified DNA was subjected to real-time PCR using the promoter-specific primers (listed below) mixed with the PowerUp SYBR Green Master Mix (Thermo Fisher Scientific) in a QuantStudio 7 Flex Real-Time PCR System (Applied Biosystems). The fast-cycling mode conditions (50 °C for 2 min, 95 °C for 2 min, 40 cycles of 95 °C for 10 s, and 60 °C for 30 s) were used to perform qPCR reactions. The rat negative control primer set 1 (#71024, Active Motif) was used as a negative control for each antibody immunoprecipitation reaction because these primers are specific for a gene desert region located on rat chromosome 3. The threshold cycle (C_T_) value in each group was normalized to the input using the following formula: (2^-ΔC^_T_) × 100%; where ΔC_T_ represents (C_T_ [ChIP] – (C_T_ [Input] - Log2 (input dilution factor). The following primers were used: *Cacna2d1* promoter (−21/+74 bp, around the transcription start site (TSS)), 5′-CGA CCG CAG ATA AAT CAG G-3′ (forward) and 5′-CAT CAA GGG CGA CTG TG-3′ (reverse); *Gapdh* (+27/+127 bp, downstream of the TSS), 5′-AGA CAG CCG CAT CTT CTT GT-3′ (forward) and 5′-CCC CCA TTC CTA ATT TCC AT-3′ (reverse).

### Statistical analysis

All data are expressed as means ± standard deviation (SD). Data collection was randomized, and the investigators conducting behavioral tests were blinded to the treatment conditions. Normal distribution for each dataset was assessed using the Shapiro–Wilk test. Statistical comparisons between two groups were made using a two-tailed Student's *t* test, whereas comparisons involving more than two groups were conducted using a two-way analysis of variance (ANOVA) followed by Tukey's *post hoc* test. All statistical analyses were performed using Prism software (version 9, GraphPad Software). A *p* value < 0.05 was considered statistically significant.

## Data availability

Data supporting the findings of this study are available from the corresponding author on reasonable request.

## Conflicts of interest

The authors declare that they have no conflicts of interest with the contents of this article.
